# Is It Possible to Improve Memory Function by Upregulation of the Cholesterol 24S-Hydroxylase (CYP46A1) in the Brain?

**DOI:** 10.1371/journal.pone.0068534

**Published:** 2013-07-16

**Authors:** Silvia Maioli, Ann Båvner, Zeina Ali, Maura Heverin, Muhammad-Al-Mustafa Ismail, Elena Puerta, Maria Olin, Ahmed Saeed, Marjan Shafaati, Paolo Parini, Angel Cedazo-Minguez, Ingemar Björkhem

**Affiliations:** 1 Department of Neurobiology, Care Sciences and Society, Karolinska Institutet-Alzheimer’s Disease Research Center, Novum, Stockholm, Sweden; 2 Department of Laboratory Medicine, Division of Clinical Chemistry, Karolinska Institutet, Karolinska University Hospital, Huddinge, Sweden; IGBMC/ICS, France

## Abstract

We previously described a heterozygous mouse model overexpressing human HA-tagged 24S-hydroxylase (CYP46A1) utilizing a ubiquitous expression vector. In this study, we generated homozygotes of these mice with circulating levels of 24OH 30–60% higher than the heterozygotes. Female homozygous CYP46A1 transgenic mice, aged 15 months, showed an improvement in spatial memory in the Morris water maze test as compared to the wild type mice. The levels of N-Methyl-D-Aspartate receptor 1, phosphorylated-N-Methyl-D-Aspartate receptor 2A, postsynaptic density 95, synapsin-1 and synapthophysin were significantly increased in the hippocampus of the CYP46A1 transgenic mice as compared to the controls. The levels of lanosterol in the brain of the CYP46A1 transgenic mice were significantly increased, consistent with a higher synthesis of cholesterol. Our results are discussed in relation to the hypothesis that the flux in the mevalonate pathway in the brain is of importance in cognitive functions.

## Introduction

Cholesterol cannot pass the blood-brain barrier and thus most or all of the cholesterol in the brain is formed by local synthesis. Under normal conditions most of the synthesis of cholesterol in the brain is balanced by formation of an oxysterol, 24S-hydroxycholesterol (24OH), which is able to pass this barrier (for reviews, see ref. [1], [2]). The enzyme responsible for formation of 24OH, the cholesterol 24S-hydroxylase, belongs to the cytochrome P-450 family, and has been given the name CYP46A1. Surprisingly, in spite of the importance of this enzyme for cholesterol homeostasis in the brain, CYP46A1 seems to be insensitive to most regulatory axes [1], [3].

Reduced activity of CYP46A1 would be predicted to result in a decreased metabolism of cholesterol with a compensatory decrease in cholesterol synthesis. It was shown that a knockout of the cyp46a1 gene in mice causes a decrease in cholesterol synthesis by about 40% [4]. The reduced cholesterol synthesis did not affect the levels of cholesterol. Overexpression of this activity would be predicted to result in an increased metabolism of cholesterol with a compensatory increase in cholesterol synthesis. In two different mouse models with overexpressed CYP46A1 the overexpression led to increased cholesterol synthesis in the brain without any changes in the levels of brain cholesterol [5], [6].

Based on the effects of 24OH on α- and β-secretase activity under in vitro conditions [7], and the assumption that increased CYP46A1 activity can be expected to decrease cholesterol content in critical membranes, we suggested that CYP46A1 activity could have a protective effect on amyloid formation in the brain [1], [8]. In accordance with this hypothesis, Hudry et al showed that local injection of an adenovirus associated vector encoding CYP46A1 in the cortex and hippocampus of a mouse model of the Alzheimeŕs disease reduced amyloid deposits in the brain and improved spatial memory [6].

In addition to the above, CYP46A1 seems to be of importance for normal memory function. A knock-out of cyp46a1 in mice resulted in severe deficiencies in spatial, associative and motor learning [9]. These changes were associated with electrophysiological changes in hippocampal slices. Treatment of such slices of wild type mice with an inhibitor of cholesterol synthesis recapitulated the effects observed in cyp46a1−/− mice. Both the genetic and the pharmacological effects could be reversed by the addition of the non-steroid isoprenoid geranyl geraniol [9]. It was hypothesized that a continuous formation of 24OH is a prerequisite for sufficient flux through the mevalonate pathway for the formation of adequate levels of isoprenoid products such as geranylgeraniol. The possibility was discussed that geranylation may be important for the function of some proteins that are critical for memory function.

In the present work we have tested the hypothesis that an increased activity of the cholesterol 24S-hydroxylase may improve memory functions in old mice.

Recently, we described a heterozygous mouse model with a stable overexpression of CYP46A1 utilizing a ubiquitous expression vector [5]. Significant levels of human CYP46A1 protein were only found in brain, testis and eye with more than 10-fold higher levels in the brain than in the other organs. The CYP46A1 protein in the brain was located to neuronal cells. It is evident that there are very specific requirements for expression of this protein in different cells. Under normal conditions CYP46A1 protein is almost exclusively expressed in neuronal cells in the brain and in the eye. It seems likely that there a number of restrictions preventing expression of this specific protein in other cells in spite of a significant expression of the corresponding mRNA.

The levels of circulating 24OH, most of which is believed to originate from the brain, were increased by a factor of 4–6 in the heterozygous mice model. The overexpression caused an increased rate of synthesis of cholesterol in the brain as shown by increased levels of cholesterol precursors [5].

We have now generated homozygotes of these mice having levels of 24OH in the circulation 30–60% higher than the heterozygotes and about 7 fold higher than the wild types. Female homozygous CYP46A1 transgenic mice, aged 15 months, showed an improvement in spatial memory in the Morris water maze test as compared to the wild type controls. The results are discussed in relation to the hypothesis that the flux in the mevalonate pathway is of importance for memory function.

## Materials and Methods

### Homozygous HA-tagged CYP46A1 Transgenic Mice

In the previous publication we characterized CYP46A1 HA-tagged heterozygous transgenic mice that had been cross-bred with C57Bl6 mice for 7 generations [5].

Heterozygous mice from the 6^th^ generation were inbred for an additional 3 generations. It was not possible to identify homozygotes with certainty by PCR only. In a typical breeding experiment the heterozygotes had copy numbers varying from 7 to 12 whereas a homozygote had a copy number of 18. The mice with the highest copy number and highest excretion of 24OH in faeces were intercrossed leading to the production of increasing number of homozygotes in the offspring. The final evidence that only homozygotes had been produced in the 8^th^ generations was obtained by crossing this generation with wildtype and finding that only heterozygotes were produced in the next generation.

Newborn female homozygous mice of the 9^th^ generation (n = 7) and corresponding newborn C57Bl6 controls (n = 7) were kept under local standard housing condition, group housed, 12 h: 12 h dark/light cycle with lights on at 08∶00, food and water ad libitum and enrichment consisting of cardboard houses and tissue paper.

### Ethics Statement

All procedures were approved by the local ethical committee of Karolinska Institutet, Stockholm (permit numbers: S135-12, S105-11 and S139-10), and performed in compliance with national and local animal care and use guidelines. All the efforts were made to minimize the suffering of the animals.

### Behavioural Tests

At fifteen months of age, animals were behaviourally tested in the elevated plus maze, open field, Morris water maze and passive avoidance tests. The sequence of the different testing steps was chosen according to the levels of stress associated with the procedures [10]. Behavioural testing was carried out between 09.00 and 15.00.

#### Open Field (OF)

Mice were placed in the experimental room 30 minutes before the test. OF activity was measured in a square arena (35×35 cm). At the beginning of a session, mice were placed in the central part of the arena, which the mouse could freely move for 30 min. Horizontal and vertical motion were detected by infrared beams and photoreceptor cells (i.e. walking was detected by horizontal beam interruptions, whereas grooming, rearing, jumping were detected by the vertical raw of IR beams). Before the first mouse and between mice, the maze was cleaned with 70% alcohol solution.

#### Elevated Plus Maze (EPM)

Mice were placed in the experimental room 30 minutes before the test. The apparatus consisted of two open arms, two enclosed arms of the same size (30×5 cm) and a central area (5×5 cm); placement was 40 cm above the floor surface. At the beginning of a session, mice were placed in the central part of the maze facing one of the open arms [11]. The number of entries and the time spent in the open and closed arms were recorded for 5 minutes. A video camera was placed above the center of the EPM and connected to a video-tracking system: Ethovision XT 8 ©. The experimenter was present in the test room during the test. Before the first mouse and between mice, the maze was cleaned with 70% alcohol solution.

#### Morris Water Maze (MWM)

Mice were trained in the MWM task to locate a hidden escape platform in a circular pool [12]. The apparatus consisted of a large circular water tank (1.40 m diameter, 70 cm height) with a transparent square escape platform (10 cm^2^). The pool was virtually divided into four equal quadrants identified as northeast, northwest, southeast, and southwest. The tank was filled with tap water at a temperature of 20±2°C up to 0.5 cm above the level of the platform and the water was made opaque with milk. The platform was placed in the tank in a fixed position (in the middle of the southwest quadrant). The pool was placed in a room with a number of extra-maze visual cues including geometric images (squares, triangles, circles) hung on the wall in addition to diffuse lighting.

Each mouse was tested for 4 trials a day, for 4 consecutive days with an inter-trial interval of 30 minutes (Acquisition). Mice were released facing the wall of the pool from one of following starting points: North, East, South or West and allowed to search the platform up to 120 seconds. For each trial, the starting position was changed. If a mouse did not find the platform, it was gently guided to it and allowed to remain there for 15 seconds. Reference memory was assessed on the fifth day, with one trial (Probe test) in which the platform was removed. A single starting point was used for all the mice. A video camera was placed above the center of the pool and connected to a video-tracking system: Ethovision XT 8 © (Noldus Information Technology B.V., Wageningen, Netherlands).

#### Passive Avoidance (PA)

The apparatus consists of two adjacent compartments: an open (devoid of ceiling) start compartment and a dark compartment. These compartments are connected by an opening, which can be obstructed manually. During the first day, mice were individually placed in the illuminated compartment. After 60-seconds of the acclimation period, the connecting door between the chambers was opened. Upon entering the dark compartment, the mice received a brief foot shock (0.3 mA for 3 seconds) and were immediately removed from the chamber. After a 24 hours retention period, the mice were placed back into the light compartment and the time to re-enter the dark compartment (latency) was measured with a threshold of 300 seconds. If the mouse entered the dark compartment before 300 seconds, no shock was administered. If the mouse remained in the light compartment for the duration of the trial (300 seconds), the door closed and the mouse was removed from the light compartment. The chambers were cleaned with 70% ethanol between testing of individual mice.

### Biochemical Measurements

2 weeks after the passive avoidance test, mice were sacrificed by cervical dislocation and brains were dissected. Western blot analysis was carried out in hippocampal tissues. Tissue homogenates were obtained as described elsewhere [13] **.** Proteins (30 ug) were separated by electrophoresis on sodium dodecyl sulfate polyacrylamide gradient gels (10%–7.5%) under reducing conditions. Membranes were probed overnight at 4°C with the following primary antibodies: rabbit anti-phospho N-Methyl-D-aspartate (NMDA) receptor 2A (1∶250, Abcam, UK), mouse anti-N-Methyl-D-aspartate receptor 1 (1∶1000, BD Bioscience, UK) and mouse anti-postsynaptic density protein 95 (PSD95) (1∶1000, Abcam, UK), rabbit anti-synapsin-1 (1∶1000, Abcam UK), mouse anti-synapthophysin (1∶1000, SY38 DakoCytomation, Denmark), mouse anti-NeuN (1∶1000, Chemicon International, CA), mouse anti-alpha-tubulin (1∶14000; Sigma Aldrich, USA). The incubation with primary antibodies was followed by incubation with anti-rabbit or anti-mouse immunoglobulin G (IgG) at 1∶10000 dilutions (Amersham Biosciences, Little Chalfont, UK). Immunoreactivity was detected by the ECL detection system (Amersham Biosciences, Little Chalfont, UK). The relative density of the immunoreactive bands was calculated from the optical density (OD) multiplied by the area of the selected band using the ImageJ 1.383 software (NIH, MA).

Brain levels of 24OH were measured by isotope dilution mass spectrometry with use of deuterium labeled internal standards as described previously [5]. Cholesterol precursors were measured by combined gas chromatography mass spectrometry as described [14].

### Statistical Analysis

Results were expressed as mean ± standard error of the mean (SEM). In the acquisition phase of the MWM, latencies to find the platform were examined by repeated measures (days) two-way ANOVA followed by HSD Tukey’s post hoc test. Probe test, passive avoidance and immunoblotting data were analysed using Mann Witney test. A value of p<0.05 was considered statistically significant.

## Results

### Characterization of Homozygous HA-tagged CYP46A1 Overexpressing Mice

**Figure 1:**
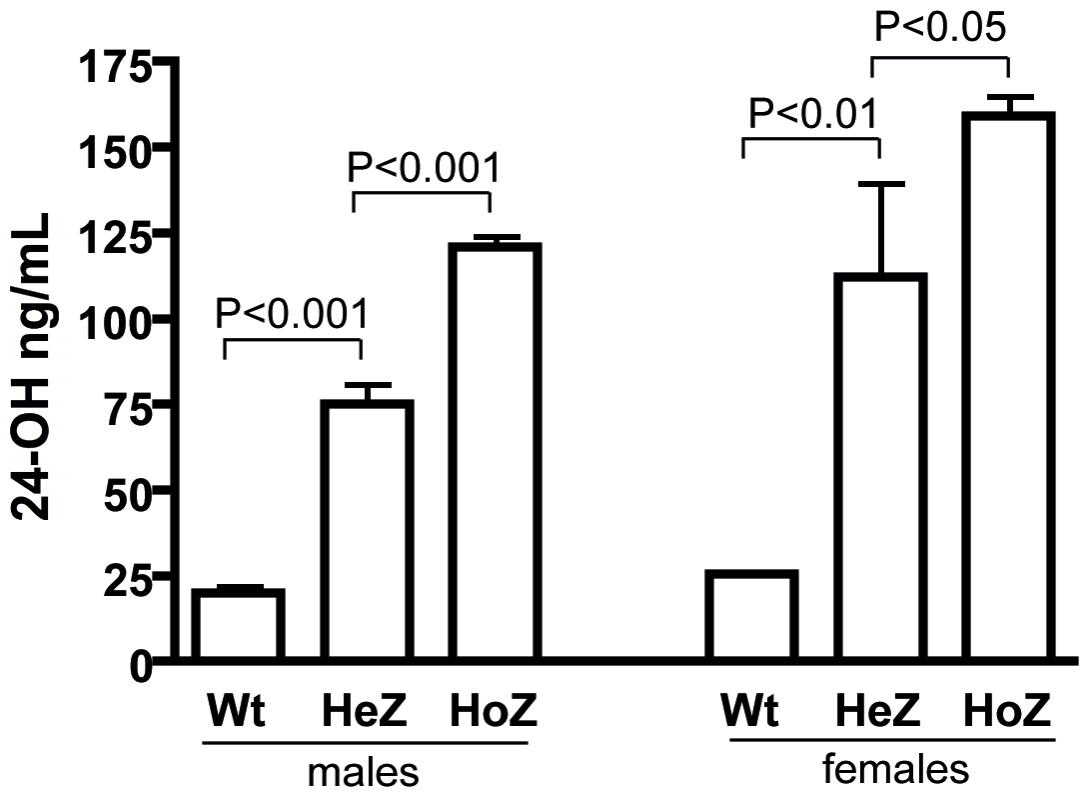
Levels of 24OH in serum from heterozygous (HeZ) and homozygous (HoZ) CYP46A1 mice (females and males) 18–21 weeks of age (n = 4–6).

As shown in Fig. 1 homozygous males and females overexpressing CYP46A1 had higher levels of 24OH in the circulation than the corresponding heterozygotes and wild type (plasma levels measured at the age of 18–21 weeks). The difference between homozygotes and heterozygotes was significant in both genders. In both male and female overexpressors, the levels of 24OH in the circulation were increased 6–7 fold when compared with wildtype. Female overexpressors had higher levels of 24OH in the circulation than male overexpressors. We also measured the mRNA levels corresponding to human CYP46A1 in the brain of male and female homozygotes. These mRNA levels were found to be almost identical in the two genders. In subsequent experiments only female overexpressors were studied.

In accordance with the results obtained previously with heterozygotes [5], the level of 24OH was significantly increased in the brain of the homozygotes (40±0 ng/mg tissue as compared to 31±1 ng/mg tissue in the controls, p<0.001).

In accordance with previous results with heterozygotes, the level of lanosterol was significantly increased in the brain of the homozygous overexpressing females (8.9±0.2 ng/mg tissue as compared to 4.7±0.2 ng/mg tissue in the controls, p<0.001). The increase in the level of lanosterol is consistent with an increased rate of synthesis of cholesterol in the brain. In accordance with the previous results obtained with heterozygotes [5], the increased synthesis was not observed at a transcriptional level. Thus the mRNA levels of HMG CoA reductase and HMG CoA synthase were not increased in the brain of the overexpressing mice as compared to the controls (p>0.05, results not shown). The level of cholesterol in the brain was not significantly different in the overexpressing mice and the control mice (p>0.05, results not shown).

### CYP46A1 Overexpression Enhances Spatial Memory Retention in Aged Female Mice

Locomotor activity and anxiety-like-behaviour was assessed in OF and EPM. No differences were found among the groups in both tests (data not shown).

MWM task was performed to assess the hippocampus-dependent spatial memory. On the first trial of the acquisition, the swimming speed of CYP46A1 and Wt mice was measured and no differences in speed or sensory motor functions (determined by visual cue test) were found between groups (data not shown). Thus, effects of motivational or sensory motor factors on learning and memory performance can be excluded. We then used the escape latency for the evaluation of spatial learning and memory of mice.

**Figure 2:**
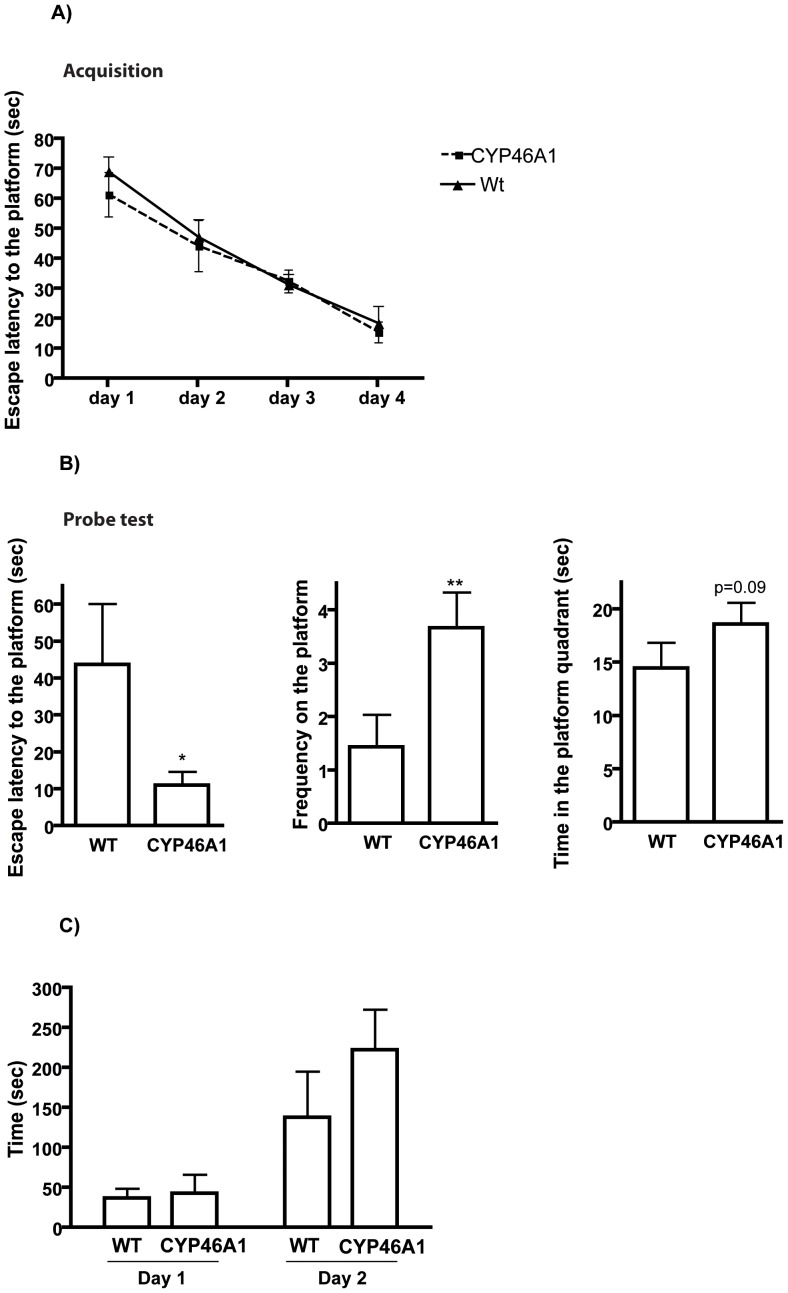
CYP46A1 overexpression enhanced spatial memory retention in aged female mice. A) Acquisition phase in MWM test: data are shown as escape latency to locate the platform over the 4 days of acquisition. B) Probe test: data are shown as escape latency time to reach the position where the platform was located, number of crossings on the platform position and the time spent in the platform target. C) Passive avoidance test: data are shown as latency time to enter the dark compartment on the training day (day 1) and the probe day (day 2) of the test. No differences were found between the groups. Results are expressed as mean ± standard error of the mean (SEM), n = 7 animal per group (**P<0.01, *P<0.05 compared to WT control mice).

Figure 2A represents the performance of 15 month old CYP46A1 and WT mice on acquisition of the MWM test. During the acquisition (from day 1 to day 4), the time to reach the hidden platform decreased over the days indicating that the animals were able to learn the task. CYP46A1 and WT mice showed similar escape latency over the days, suggesting that the spatial learning was not affected in any of the groups. Interestingly, when only the first trial per day was analyzed we could observe that CYP46A1 presented shorter escape latency (8.24±2.1 sec) than the WT (29.81±11.1 sec) at day 4 (*p<0.05, data not shown). This result suggests that on day 4 CYP46A1 mice were able to retain the spatial information from the day before and therefore reached the position of the platform in shorter time than the WT during the first trial. This improvement in memory retention is further confirmed in the probe test as described below.

On the fifth day of the test, all mice were subjected to a probe trial in which the platform was removed. Figure 2B shows the performance of the mice during the probe test. As measures of retention memory we used: the time of first occurrence to the position where the platform was located during acquisition, the number of times that animals crossed the former platform area and the time spent in the target quadrant of the platform. Escape latencies of CYP46A1 mice were significantly shorter than those of WT group (F = 50.89, P<0.05, n = 7). Moreover, CYP46A1 animals performed higher number of crossover on the platform as compared to WT animals (F = 1.667, P<0.01, n = 7). No difference was found in the time spent in the quadrant where the platform was located, although a tendency towards significance (P = 0.09) was found. These data suggest that the overexpression of CYP46A1 enhances spatial memory retention in aged mice.

Emotional learning and memory were assessed in a passive avoidance test. Figure 2C shows the latency time to enter the dark compartment on the first day (training) and on the second day (probe) of the test. On the first day, all the groups showed similar step-through latencies, indicating no differences in locomotor activity and anxiety-like behaviour among the groups. After 24 hours (day 2), animals were re-placed in the test apparatus to test their memory. During this second day, no significant differences in the latency time to re-enter the dark compartment were found among the groups, indicating that this type of memory was not affected by the overexpression of CYP46A1.

### Levels of Synaptic Proteins were Increased in the Hippocampus of CYP46A1 Mice

To explore whether CYP46A1 mice exhibit biochemical modifications consistent with the improvement observed in spatial memory retention, we investigated the levels of synaptic proteins in hippocampal lysate by western blotting.

**Figure 3:**
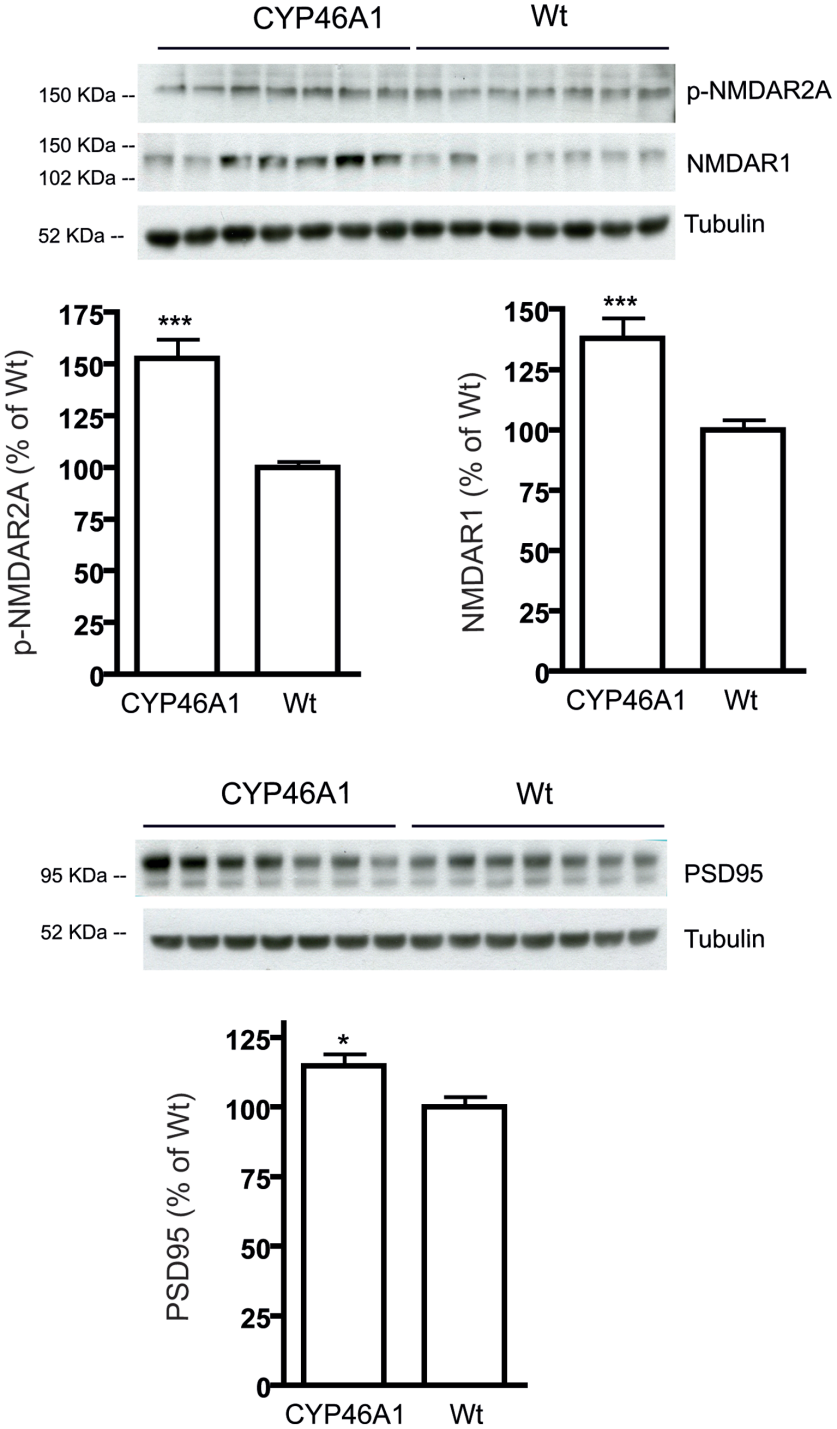
Levels of postsynaptic proteins were increased in the hippocampus of CYP46A1 mice. Hippocampal samples from CYP46A1 and WT control mice were analyzed by Western blotting. NMDAR1, p-NMDAR2A and PSD95 levels were significantly increased in CYP46 mice. Data are shown as mean ± standard error of the mean (SEM) of immunoreactivity (OD x area of the band) normalizes by α-tubulin levels. N = 7 animal per group (***P<0.001, *P<0.01 compared to WT control mice).

We first analyzed postsynaptic proteins, such as N-Methyl-D-Aspartate receptors (NMDAR) subunits (NMDAR1 and p-NMDAR2A). NMDAR are located in the post-synaptic membrane and are the primary mediators of excitatory synaptic transmission in the hippocampus. As shown in Figure 3, levels of NMDAR1 and p-NMDAR2A were significantly increased in 15 months old CYP46A1 mice when compared to WT mice (NMDAR1: F = 4.665, P<0.001, n = 7; p-NMDAR2A: F = 9.4, P<0.001, n = 7). Analysis of Post synaptic density 95 (PSD95), a scaffolding protein involved in the assembly and the function of the postsynaptic density complex, revealed that CYP46A1 mice had significant higher levels in the hippocampus as compared with WT mice (F = 1.271, P<0.05, n = 7).

**Figure 4:**
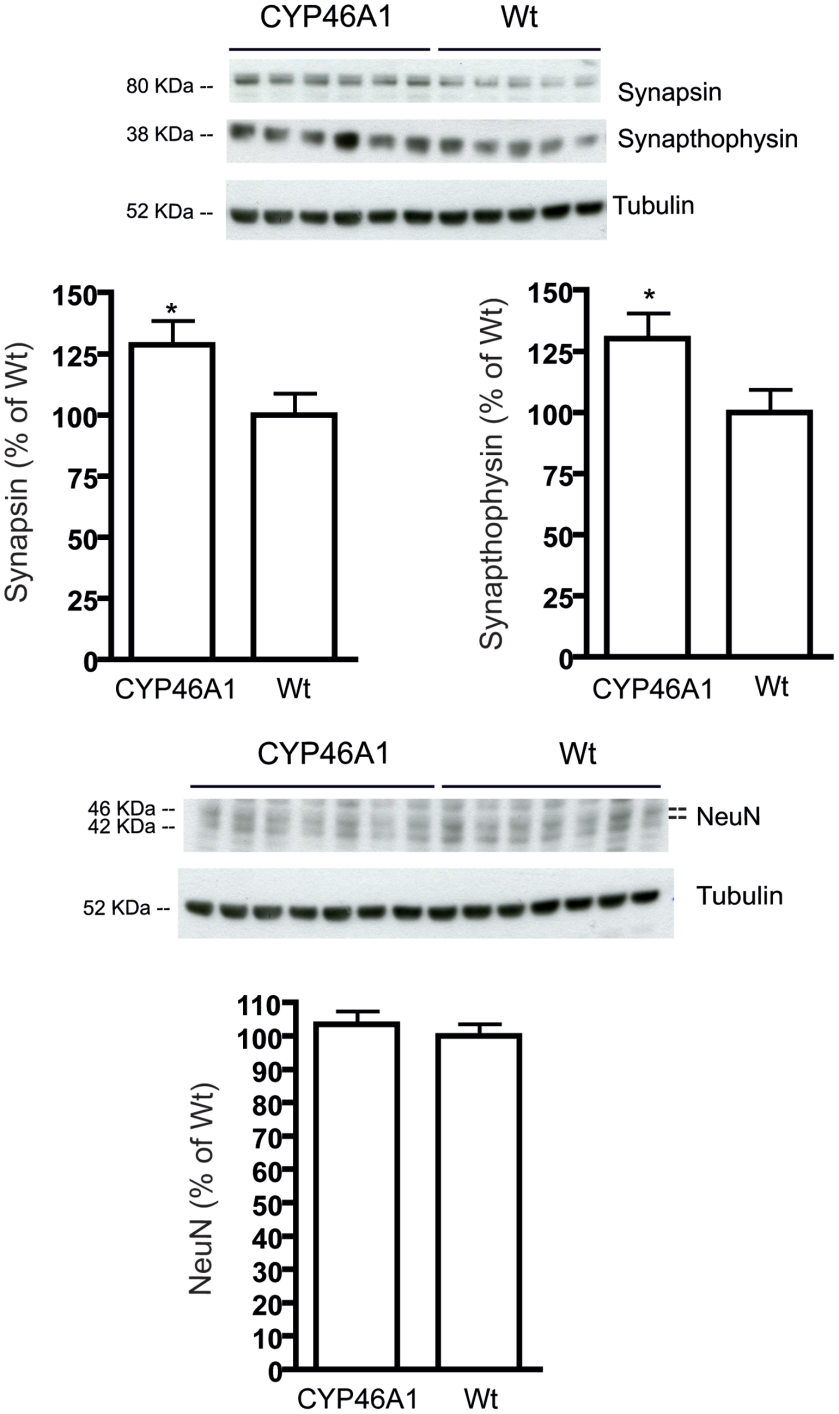
Levels of presynaptic proteins were increased in the hippocampus of CYP46A1 mice. Hippocampal samples from CYP46A1 and WT control mice were analyzed by Western blotting. Synapthophysin and synapsin-1 levels were significantly increased in CYP46A1 mice, while no differences were found in Neun among the groups. Data are shown as mean ± standard error of the mean (SEM) of immunoreactivity (OD x area of the band) normalizes by α-tubulin levels. N = 7 animal per group (*P<0.01 compared to WT control mice).

We next investigated presynaptic proteins, such as synapthophysin and synapsin-1. Synapthophysin is the most abundant synaptic vesicle protein and is often used as a marker for quantifying the number of intact synapses. Synapsin-1 is another presynaptic protein that regulates neurotransmitter release. As shown in Figure 4, synapthophysin and synapsin-1 levels are increased in CYP46A1 mice as compared with WT mice (synapthophysin: F = 1.282 P<0.05, n = 7; synapsin-1: F = 1.249, P<0.05, n = 7). Analysis of NeuN, a neuronal marker, revealed no differences between groups.

## Discussion

Overexpression of CYP46A1 in a mouse model with increased tendency to accumulate β-amyloid in the brain was shown to decrease amyloid deposition and to improve cognition [6]. Whether most or all of the beneficial effect of the overexpression on cognition found in that study is secondary to the reduced burden of β-amyloid or whether part of it is due to the increased flux in the mevalonate pathway is not possible to evaluate. In view of the *in vitro* effects of 24OH on the generation of β-amyloid, the beneficial effects demonstrated may be due to an effect of the increased levels of 24OH in the brain rather than due to the increased flux in the mevalonate pathway. The hypothesis tested here is that overexpression of CYP46A1 is also able to improve memory function in aged mice without a genetic tendency to accumulate amyloid.

To this aim, we tested CYP46A1 mice and their wild type controls at an advanced age, 15 months old, in spatial and non-spatial memory tests. The homozygous CYP46A1 overexpressing mice studied here showed slightly higher levels of 24OH in the circulation, and similar increases in cholesterol precursors in the brain than the previously described heterozygotes [5]. This was also seen in mice overexpressing CYP27 using the same promoter [15], where both homozygotes and heterozygotes shared similar biochemical characteristics.

Our data showed that overexpression of CYP46A1 had a positive effect on spatial memory retention in MWM test. This observed enhancement of cognitive functions occurred in parallel with increased hippocampal levels of postsynaptic and presynaptic markers such as NMDAR1, p-NMDAR2A, PSD95, synapthophysin and synapsin-1. NMDA receptors, generally anchored in the postsynaptic density, are required for hippocampal synaptic plasticity as well as spatial learning and memory [16], [17], [18]. Y1325 phosphorylated NMDAR2A subunit is considered a good indicator of NMDAR activity [13]. It has been reported that loss of functional subunits, such as NMDAR1 and NMDAR2A would indicate a deficit or alteration in synaptic function. Studies in aging rodents have shown that functional impairments of these receptors are associated with spatial learning and memory deficits [19], [20], [21]. Also reduction in expression and levels of presynaptic proteins, such as synapthophysin and synapsin-1, was reported in aging hippocampus and various cortical structures of human and rodents [22], [23], [24]. According to these findings, our data suggest that the enhancement of spatial memory processes may be linked with the increased levels of synaptic proteins in hippocampus, suggesting a protective effect of CYP46A1 on cognitive functions in normal aging processes.

In spite of the increased levels of synaptic proteins the levels of cholesterol in the brain of the CYP46A1 transgenic mice were not different from those of the controls. Consistent with our previous studies with heterozygotes, evidence showed that the overexpression of CYP46A1 is associated with an increased rate of synthesis of cholesterol (increased levels of the cholesterol precursor lanosterol). This effect was rather moderate, however the increased synthesis could not be observed at the transcriptional level. Thus, it is tempting to suggest that there may be a relation between cognitive function and flux in the mevalonate pathway not only at a low rate of this flux as shown previously [6] but also at an increased rate of this flux. It is noteworthy that cholesterol synthesis is reduced in Huntingtońs disease (for a review see ref. [25]). The situation in Alzheimeŕs disease (AD) is controversial. In AD, it has been shown that β-amyloid reduces cholesterol synthesis but also that increased levels of cholesterol in critical membranes may increase production of β-amyloid (for a review see ref. [25]).

If there is a relation between cognition and rate of brain cholesterol synthesis as suggested by the work by Kotti et al [9] and the present study, this could have clinical implications. For example, inhibition of cholesterol synthesis by statins would not be expected to have a beneficial effect on cognition. Indeed, loss of memory has been reported to be a rare side-effect of treatment with statins [26]. In summary, our results suggest that increased levels of 24OH have beneficial effects on cognition and synaptic plasticity in old female mice. Further work is needed, however, to confirm a relation between cholesterol synthesis in the brain and cognitive function.
